# Angiopoietins, Vascular Endothelial Growth Factors and Secretory Phospholipase A_2_ in Ischemic and Non-Ischemic Heart Failure

**DOI:** 10.3390/jcm9061928

**Published:** 2020-06-19

**Authors:** Gilda Varricchi, Stefania Loffredo, Leonardo Bencivenga, Anne Lise Ferrara, Giuseppina Gambino, Nicola Ferrara, Amato de Paulis, Gianni Marone, Giuseppe Rengo

**Affiliations:** 1 Department of Translational Medical Sciences, University of Naples Federico II, 80100 Naples, Italy; leonardobencivenga@gmail.com (L.B.); Anneliseferrara@gmail.com (A.L.F.); pina.gambino@gmail.com (G.G.); nicferra@unina.it (N.F.); depaulis@unina.it (A.d.P.); marone@unina.it (G.M.); giuseppe.rengo@unina.it (G.R.); 2 Center for Basic and Clinical Immunology Research (CISI), University of Naples Federico II, 80100 Naples, Italy; 3 World Allergy Organization (WAO), Center of Excellence, 80100 Naples, Italy; 4 Institute of Experimental Endocrinology and Oncology “G. Salvatore” (IEOS), National Research Council (CNR), 80100 Naples, Italy; 5 Department of Advanced Biomedical Sciences, University of Naples Federico II, 80100 Naples, Italy; 6 Istituti Clinici Scientifici Maugeri SpA Società Benefit, Via Bagni Vecchi, 1, 82037 Telese BN, Italy

**Keywords:** angiopoietins, heart failure, VEGFs, sPLA_2_, IHF, NIHF

## Abstract

Heart failure (HF) is a growing public health burden, with high prevalence and mortality rates. In contrast to ischemic heart failure (IHF), the diagnosis of non-ischemic heart failure (NIHF) is established in the absence of coronary artery disease. Angiopoietins (ANGPTs), vascular endothelial growth factors (VEGFs) and secretory phospholipases A_2_ (sPLA_2_s) are proinflammatory mediators and key regulators of endothelial cells. In the present manuscript, we analyze the plasma concentrations of angiogenic (ANGPT1, ANGPT2, VEGF-A) and lymphangiogenic (VEGF-C, VEGF-D) factors and the plasma activity of sPLA_2_ in patients with IHF and NIHF compared to healthy controls. The concentrations of ANGPT1, ANGPT2 and their ratio significantly differed between HF patients and healthy controls. Similarly, plasma levels of VEGF-D and sPLA_2_ activity were higher in HF as compared to controls. Concentrations of ANGPT2 and the ANGPT2/ANGPT1 ratio (an index of vascular permeability) were increased in NIHF patients. VEGF-A and VEGF-C concentrations did not differ among the three examined groups. Interestingly, VEGF-D was selectively increased in IFH patients compared to controls. Plasma activity of sPLA_2_ was increased in IHF and NIHF patients compared to controls. Our results indicate that several regulators of vascular permeability and smoldering inflammation are specifically altered in IHF and NIHF patients. Studies involving larger cohorts of these patients will be necessary to demonstrate the clinical implications of our findings.

## 1. Introduction

Heart failure (HF) represents a growing public health burden with an estimated prevalence in Europe and United States ranging from 0.4% to 2% [1]. Based on left ventricle ejection fraction (EF), HF recognizes three different classes: HF with a reduced EF (HFrEF with an EF < 40%); HF with a mild-range EF (HFmEF with an EF between 40% and 49%), and HF with a preserved EF (HFpEF with an EF ≥ 50%) [2]. Although classification systems for HF causes are largely debated, within HFrEF ischemic heart disease represents the most common cause of myocardial injury and ventricular dysfunction, leading in a significant percentage of cases to post-ischemic heart failure (IHF). Non-ischemic HF (NIHF), which accounts for less than 50% of HFrEF cases, comprises all the remaining heterogeneous HF etiologies ranging from valvular diseases to toxic damage, up to metabolic conditions and genetic cardiomyopathies [3]. In a significant percentage (≅ 30%) of HF patients, the etiology remains undetermined, and the syndrome is referred to as “idiopathic HF” [4]. Identification of these diverse etiologies may be obtained through a complex diagnostic workup, frequently without a relevant therapeutic implication. Neurohormonal and inflammatory activation are widely recognized as playing a pivotal role in HF onset and progression, irrespective of etiology [5]. Despite advances in management and therapies, the prognosis in HF patients remains poor, thus a deeper knowledge of the molecular mechanisms involved in the complex HF pathophysiology are needed for the identification of novel therapeutic targets and biomarkers to stratify prognosis and drive decision-making processes [6]. To this aim, several investigations have focused their attention on inflammatory and neurohormonal molecules.

The angiopoietin (ANGPT) family is an important group of factors, specific for vascular endothelium, whose functions are mediated through two tyrosine kinase receptors, Tie1 and Tie2 [7]. The ANGPT-Tie ligand-receptor system exerts a key role in regulating vascular integrity [8,9]. Beside their roles in the modulation of angiogenesis [10,11] and lymphangiogenesis [12,13], ANGPTs also regulate inflammation in several disorders, including cardiovascular diseases [9,14,15]. Angiopoietin-1 (ANGPT1), produced by peri-endothelial mural cells (pericytes) [16] and immune cells [17,18], is a potent agonist of Tie2 receptor on endothelial cells [11,19]. ANGPT1 is an anti-inflammatory molecule [20] that maintains vascular integrity [21,22]. ANGPT2, stored in Weibel–Palade bodies in endothelial cells [23], is rapidly released in response to various stimuli [24]. ANGPT2 is considered a pro-inflammatory molecule [25,26] and inhibits ANGPT1/Tie2 interaction [10,27], resulting in vascular instability and leakage [26].

Elevated ANGPT2 levels have been found in patients with acute coronary syndrome [28,29], hypertension [30,31], congestive heart failure [32] and congenital heart failure [33]. ANGPT2 has been proposed as a prognostic biomarker of adverse cardiovascular events in myocardial infarction [34] and after percutaneous coronary intervention (PCI) [35,36]. In contrast, ANGPT1 plays a protective role in rodent models of vascular injuries [37,38].

The vascular endothelial growth factor (VEGF) family includes VEGF-A, VEGF-B, VEGF-C, and VEGF-D [39]. VEGFs and their receptors on blood and lymphatic endothelial cells play intricate roles in initiating and promoting inflammatory and tumor angiogenesis [40]. VEGF-A, the most potent proangiogenic factor [41], was first identified for its permeabilizing activity and named vascular permeability factor (VPF) [42]. VEGF-A and VEGF-B are key regulators of systemic and cardiac angiogenesis [39,43,44]. VEGF-C and VEGF-D are the most important modulators of inflammatory and tumor lymphangiogenesis [45,46]. Several studies have found elevated levels of circulating VEGF-A in patients with myocardial infarction [28,47,48,49,50]. By contrast, the roles of VEGF-A [32] VEGF-C and VEGF-D in HF remain unclear or totally unexplored.

Phospholipases A_2_ (PLA_2_) hydrolyze the fatty acids from membrane phospholipids releasing arachidonic acid and lysophospholipids [51,52,53,54]. Secreted or extracellular PLA_2_ (sPLA_2_) modulate vascular permeability [55] and activate inflammatory cells [53,56,57]. Circulating levels of sPLA_2_ predict coronary events in patients with coronary artery disease [58] and in apparently healthy men and women [59]. Serum sPLA_2_ levels also predict long-term mortality for HF after myocardial infarction [60]. Intima of coronary atherosclerotic lesions of patients with angina or myocardial infarction express sPLA_2_ [61] and elevated serum levels of sPLA_2_ increase the risk of early atherosclerosis [62].

While some studies are available on ANGPTs, VEGF isoforms, and sPLA_2_ involvement in ischemic heart disease, very little is known in the clinical setting of IHF and, to the best of our knowledge, no data are available in NIHF. Thus, the aim of the present study is to evaluate the circulating levels of ANGPTs, VEGFs, and sPLA_2_ activity in HF patients, particularly comparing the ischemic and non-ischemic etiologies.

## 2. Materials and Methods

### 2.1. Study Population

Patients with systolic HF were enrolled at the Department of Translational Medical Sciences of the University of Naples Federico II. Inclusion criteria were: age ≥ 18 years, diagnosis of HF from at least six months [2], left ventricular ejection fraction (LVEF) ≤ 45%, stable clinical condition during the month prior to inclusion, and an optimal guideline-based pharmacotherapy from at least three months, if not contraindicated. Exclusion criteria were represented by chronic obstructive pulmonary disease (COPD), diabetes mellitus (DM), immune disorders (rheumatoid arthritis, systemic lupus erythematosus, systemic sclerosis, Sjögren syndrome, vasculitis, psoriatic arthritis, dermatomyositis, ankylosing spondylitis), malignancies (also past), severe obesity as assessed through a body mass index (BMI) more than 32 kg/m^2^, dialysis-dependent kidney failure, acute coronary syndromes and/or coronary revascularization in the previous 6 months, and an inability to provide informed consent. The control group was represented by subjects without HF and in accordance with the exclusion criteria. All patients underwent medical history evaluation and collection of demographic/clinical data, including age, gender, BMI, cardiovascular risk factors, and comorbidities. Clinical examination, transthoracic echocardiography, and serum BNP determination were performed at the time of the enrolment. The HF population was subsequently divided into two groups based on the HF etiology: ischemic HF (IHF) or non-ischemic HF (NIHF). Ischemic etiology was established based on either previous documented myocardial infarction and/or significant coronary artery disease with indication of cardiac revascularization. This study was approved by the Ethics Committee of the University of Naples Federico II (protocol number 124/17). All participants were carefully informed and signed a written consent to participate in the study.

### 2.2. Blood Sampling

Blood was collected during routine diagnostic procedures, scheduled in the course of hospital access for the determination of the main blood parameters (blood counts, biochemical. and coagulation profile), and the remaining plasma sample was labeled with a code that was documented into a data sheet. As mentioned above, blood samples were collected in patients under stable clinical conditions, strictly verifying all inclusion and exclusion criteria. The samples were collected by means of a clean venipuncture and minimal stasis using sodium citrate 3.2% as anticoagulant. After centrifugation (2000 g for 20 min at 22 °C), the plasma was divided into aliquots and stored at −80 °C until used. Technicians who performed the assays were blinded to the patients’ history.

### 2.3. Assays of ANGPTs and VEGFs

Plasma levels of ANGPT1, ANGPT2, VEGF-A, VEGF-C, and VEGF-D were measured using commercially available ELISA kits (R&D System, Minneapolis, MN, USA) according to the manufacturer’s instructions. The ELISA sensitivity was 156.25–10,000 pg/mL for ANGPT1, 31.1–4000 pg/mL for ANGPT2, 31.1–2000 pg/mL for VEGF-A, 62.5–4000 pg/mL for VEGF-C, and 31.3–2000 pg/mL for VEGF-D.

### 2.4. Assay of Phospholipase A_2_ Activity

PLA_2_ activity in the plasma of patients and healthy controls was measured by Life Technologies EnzChek (Milan, Italy) phospholipase A_2_ assay. Briefly, a PLA_2_ substrate cocktail consisting of 7-hydroxycoumarinyl-arachidonate (0.3 mM), 7-hydroxycoumarinyl-linolenate (0.3 mM), hydroxycoumarinyl 6-heptenoate (0.3 mM), dioleoylphosphatidylcholine (DOPC) (10 mM), and dioleoylphosphatidylglycerol (DOPG) (10 mM) was prepared in ethanol. Liposomes were formed by gradually adding 77 µL substrate/lipid cocktail to 10 mL of PLA_2_ buffer (50 mM Tris–HCl, 100 mM NaCl, 1 mM CaCl_2_) while stirring rapidly over 1 min using a magnetic stirrer. Fluorescence (excitation at 360 nm and emission at 460 nm) was measured and specific activity [relative fluorescent units (RFU)/mL] for each sample was calculated. Plasma (50 µL) was added to 96-well plates, and PLA_2_ activity was evaluated by adding 50 µL of substrate cocktail.

### 2.5. Statistical Analysis

The sample size was determined by the primary outcome, which was defined through a comparison of ANGPT2 plasma levels between HF patients and healthy controls in a 1:1 ratio. Assuming an alpha error equal to 5% and a statistical power equal to 80%, considering the mean concentrations of ANGPT2 to be approximately 500 pg/mL in healthy individuals, according to previous evidence [32], a minimum of 70 patients (35 per group) are necessary to capture as significant a 40% difference in ANGPT2 plasma concentration between controls and HF patients. Data were analyzed with the GraphPad Prism 7 software package. Data were tested for normality using a D’Agostino-Pearson normality test. If normality was not rejected at the 0.05 significance level, we used parametric tests. Otherwise, for not-normally distributed data we used nonparametric tests. Statistical analysis was performed using a Student’s *t*-test or one-way ANOVA and Bonferroni’s multiple comparison test, as indicated in the figure legends. Correlations between two variables were assessed by Spearman’s rank correlation analysis and reported as coefficients of correlation (*r*). Plasma concentrations of VEGFs and ANGPTs and activity of sPLA_2_ are shown as the median (horizontal black line), the 25th and 75th percentiles (boxes), and the 5th and 95th percentiles (whiskers) of HF, NIHF, and IHF patients and controls. Statistically significant differences were accepted when the *p*-value was ≤0.05.

## 3. Results

### 3.1. Clinical and Demographic Characteristics of Overall Population

Table 1 summarizes the demographic and clinical characteristics of patients with IHF, NIHF, and matched healthy controls. The overall study population comprised 43 patients suffering from HF and 42 healthy donors, carefully selected according to inclusion/exclusion criteria. Patients with HF were divided into two groups based on HF etiology [3]: 19 with IHF and 25 with NIHF. Both HF groups were homogeneous in age, gender, BNP levels and LVEF. As expected, IHF and NIHF showed higher BNP levels and lower LVEFs compared to healthy controls (Table 1).

### 3.2. Plasma Concentrations of ANGPT1, ANGPT2, VEGF-A, VEGF-C, VEGF-D and PLA_2_ Activity in Healthy Controls and HF Patients

**Figure 1:**
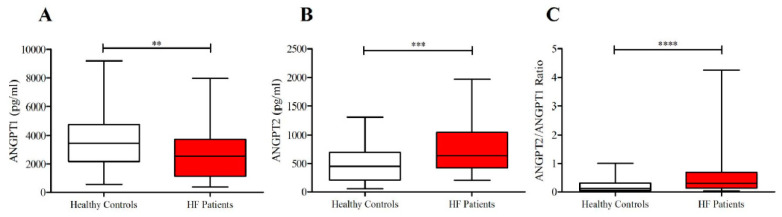
(**A**) Plasma concentrations of angiopoietin-1 (ANGPT1) in heart failure (HF) patients and in healthy controls; (**B**) Plasma concentrations of ANGPT2 in HF patients and in healthy controls; (**C**) ANGPT2/ANGPT1 ratio in HF patients and in healthy controls. Data are shown as the median (horizontal block line), the 25th and 75th percentiles (boxes), and the 5th and 95th percentiles (whiskers) (statistical analysis was performed by a Student’s *t*-test). ** *p* < 0.01; *** *p* < 0.001; **** *p* < 0.0001.

**Figure 2:**
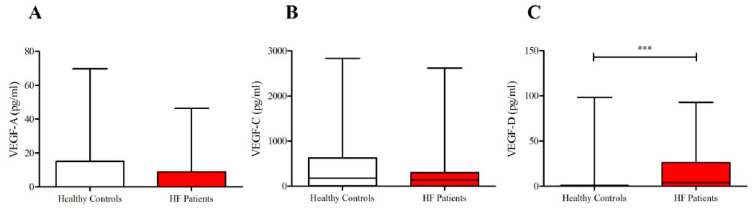
(**A**) Plasma concentrations ofvascular endothelial growth factor-A (VEGF-A) in heart failure (HF) patients and in healthy controls; (**B**) plasma concentrations of VEGF-C in HF patients and in healthy controls; (**C**) plasma concentrations of VEGF-D in HF patients and in healthy controls. Data are shown as the median (horizontal block line), the 25th and 75th percentiles (boxes), and the 5th and 95th percentiles (whiskers) (statistical analysis was performed by a Student’s *t*-test). *** *p* < 0.001.

**Figure 3:**
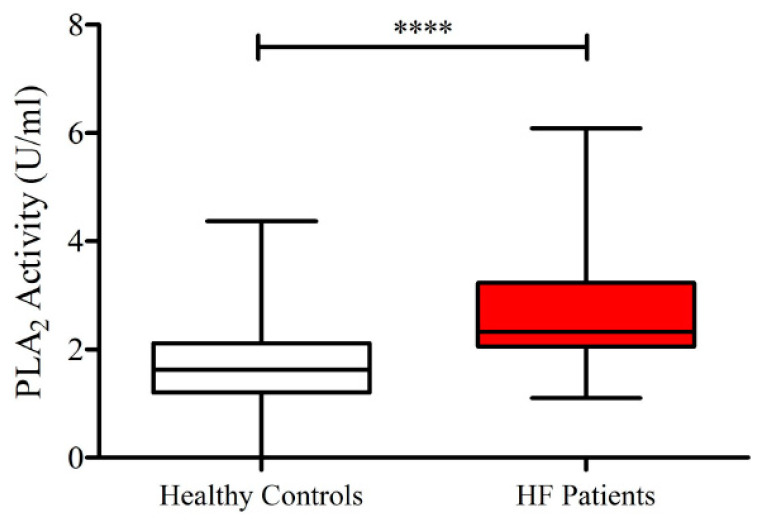
Plasma concentrations of sPLA_2_ activity in HF patients and in healthy controls. Data are shown as the median (horizontal block line), the 25th and 75th percentiles (boxes), and the 5th and 95th percentiles (whiskers) (statistical analysis was performed by a Student’s *t*-test). **** *p* < 0.0001.

As shown in Figure 1, lower concentrations of ANGPT1 and higher levels of ANGPT2 and ANGPT2/ANGPT1 ratios were detected in subjects suffering from HF compared to healthy controls. No differences were observed in plasma concentrations of VEGF-A and VEGF-C in the two groups (Figure 2). Otherwise, HF patients presented higher concentrations of VEGF-D compared to controls. Moreover, HF was associated with higher PLA_2_ activity (Figure 3).

### 3.3. Plasma Concentrations of ANGPT1, ANGPT2 and Their Ratio in Patients With IHF and NIHF

**Figure 4:**
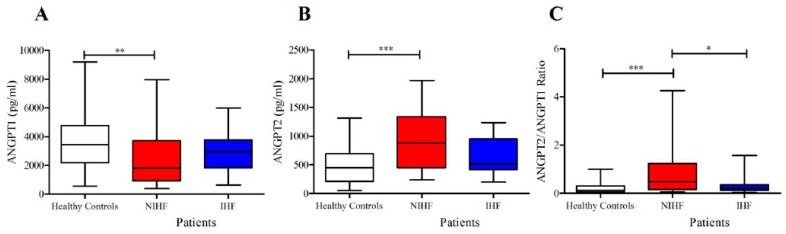
(**A**) Plasma concentrations of angiopoietin-1 (ANGPT1) in ischemic (IHF) and non-ischemic (NIHF) patients, and in healthy controls; (**B**) plasma concentrations of ANGPT2 in IHF and NIHF patients, and in healthy controls; (**C**) ANGPT2/ANGPT1 ratio in IHF and NIHF patients, and in healthy controls. Data are shown as the median (horizontal block line), the 25th and 75th percentiles (boxes), and the 5th and 95th percentiles (whiskers) (statistical analysis was performed by one-way ANOVA and Bonferroni’s multiple comparison test). * *p* < 0.05; ** *p* < 0.01; *** *p* < 0.001.

The concentrations of ANGPT1 were significantly reduced in NIHF compared to controls (Figure 4A). By contrast, the plasma concentrations of ANGPT2 were selectively increased only in NIHF compared to healthy donors (Figure 4B). Similarly, the ANGPT2/ANGPT1 ratio, a parameter of vascular permeability [63], was also increased only in NIHF patients compared to controls (Figure 4C). Importantly, no difference emerged between IHF group and healthy controls in the ANGPT2/ANGPT1 ratio, whereas there was a significant difference between the ANGPT2/ANGPT1 ratio in NIHF vs. IHF (Figure 4C). There were no differences in ANGPT1 or ANGPT2 between male and female values in both controls and patients. Moreover, the age of patients and the concentrations of the different mediators examined did not correlate.

### 3.4. Plasma Concentrations of VEGF-A, VEGF-C, and VEGF-C in Patients with IHF and NIHF

**Figure 5:**
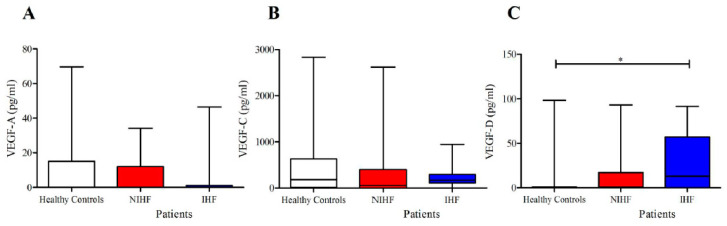
(**A**) Plasma concentrations of VEGF-A in IHF and NIHF patients and in healthy controls; (**B**) plasma concentrations of VEGF-C in IHF and NIHF patients and in healthy controls; (**C**) plasma concentrations of VEGF-D in IHF and NIHF patients and in healthy controls. Data are shown as the median (horizontal block line), the 25th and 75th percentiles (boxes), and the 5th and 95th percentiles (whiskers) (statistical analysis was performed by one-way ANOVA and Bonferroni’s multiple comparison test). * *p* < 0.05

VEGF-A is a powerful permeability [42] and angiogenic mediator [41]. Elevated concentrations of VEGF-A have been found in patients with acute myocardial ischemia [28,47,48,49,50]. By contrast, the role of VEGF-A in chronic heart failure remains unclear [32]. We found that the mean plasma concentrations of VEGF-A were essentially similar in patients with different types of HF and controls (Figure 5A).

VEGF-C and VEGF-D are known to play a major role as lymphangiogenic factors acting on VEGF receptor 3 (VEGFR3) on lymphatic endothelial cells (LECs) [64,65]. More recently, it has been shown that these factors are produced by human cardiac mast cells [43] and, under certain circumstances, can exert a protective effect in cardiovascular disorders [66,67]. In addition, it has been demonstrated that VEGF-C and VEGF-D can exert different effects [45]. The mean plasma concentrations of VEGF-C did not differ in patients with different HF types and controls (Figure 5B). In contrast, the plasma concentrations of VEGF-D were increased in IHF patients compared to healthy controls (Figure 5C). There were no differences in VEGF-A, VEGF-C, and VEGF-D concentrations between male and female values in either controls and patients. Moreover, the age of patients and the concentrations of VEGFs examined did not correlate.

### 3.5. Plasma Concentrations of sPLA_2_ Activity in Patients With IHF and NIHF

**Figure 6:**
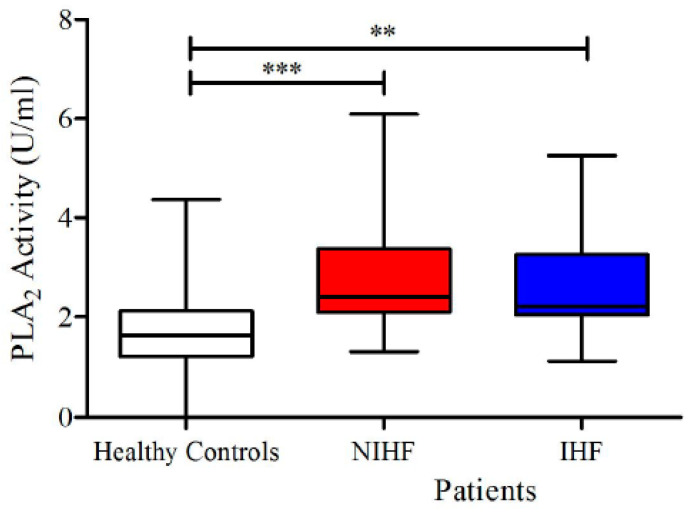
Plasma concentrations of sPLA_2_ activity in IHF and NIHF patients and in healthy controls. Data are shown as the median (horizontal block line), the 25th and 75th percentiles (boxes), and the 5th and 95th percentiles (whiskers) (statistical analysis was performed by one-way ANOVA and Bonferroni’s multiple comparison test). ** *p* < 0.01; *** *p* < 0.001.

sPLA_2_ modulates vascular permeability [55] and promotes inflammation [52,53,56]. Circulating sPLA_2_ levels increase the risk of early atherosclerosis [62] and predict long-term mortality of HF after myocardial infarction [60]. Figure 6 shows that plasma activity of sPLA_2_ activity was significantly increased in both groups of HF patients compared to healthy controls. There was no differences in sPLA_2_ activity between male and female values in both controls and patients. Moreover, the age of patients and the concentration of sPLA_2_ activity did not correlate.

### 3.6. Correlations between ANGPT1 or ANGPT2 Plasma Concentrations and sPLA_2_ Activity in Patients with IHF and NIHF

**Figure 7:**
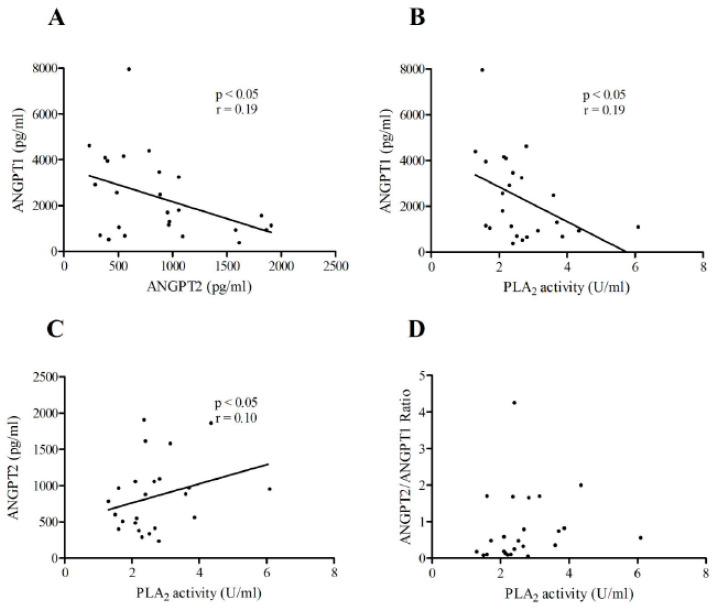
(**A**) Correlations between the plasma concentrations of ANGPT2 and ANGPT1 in NIHF patients; (**B**) correlation between circulating sPLA_2_ activity and the concentration of ANGPT1 in NIHF patients; (**C**) correlation between the plasma concentration of sPLA_2_ activity and ANGPT2 in NIHF patients; (**D**) correlation between the plasma concentration of sPLA_2_ activity and the ANGPT2/ANGPT1 ratio in NIHF patients. Spearman’s correlation coefficients (*r*) were calculated and are shown in the panels.

As shown in Figure 7, there was an inverse correlation between plasma concentrations of ANGPT2 and ANGPT1 (Figure 7A) and sPLA_2_ activity and ANGPT1 (Figure 7B) in NIHF patients. Furthermore, a positive correlation between PLA_2_ activity and ANGPT2 was detected in NIHF (Figure 7C). No correlation was observed between sPLA_2_ activity and the ANGPT2/ANGPT1 ratio in NIHF.

Contrariwise, no correlations were observed among the plasma concentrations of ANGPT1 and BNP, ANGPT2 and BNP, and sPLA_2_ activity and BNP in NIHF patients. Similarly, no correlations were found between plasma concentrations of ANGPT1, ANGPT2, and sPLA_2_ activity vs. LVEF in patients with IHF or NIHR.

## 4. Discussion

To the best of our knowledge, this is the first study reporting significant and distinct alterations of plasma concentrations from three different classes of proinflammatory mediators that are essential for vascular development, integrity and remodeling (i.e., angiopoietins, VEGFs, and secretory phospholipase A_2_) in patients with two forms of HF (i.e., ischemic and non-ischemic).

ANGPTs bind to and activate the Tie2 receptor on endothelial cells [9,27]. ANGPT1, produced by periendothelial mural cells [16] acts as a vascular stabilizer by affecting the connections between endothelial cells and the cytoskeleton [68]. In contrast, ANGPT2, produced by blood endothelial cells [23], is rapidly released from Wiebel–Palade bodies in response to various stimuli [24]. ANGPT2 also binds to Tie2 [27] and antagonizes ANGPT1-mediated Tie2 phosphorylation, thereby inducing vascular instability and leakage [25,26,69]. In addition, ANGPT2 is an important permeability [63,70] and proinflammatory mediator [16].

Elevated circulating levels of ANGPT2 have been reported in acute coronary syndromes [28,29], and this mediator has been proposed as a negative prognostic marker after myocardial infarction [34] and PCI [35,36]. ANGPT2 is associated with a greater risk of cardiovascular mortality in the general population [71], as well as with higher mortality in patients suffering from myocardial infarction and cardiogenic shock [29,72]. A recent report demonstrates that ANGPT2 is highly expressed in endothelial cells at the border of the infarct area after ischemic injury in mice [15]. In the remodeling phase after myocardial infarction, endothelial- and macrophage-derived ANGPT2 promotes abnormal vascular remodeling and exacerbates inflammation. In contrast, ANGPT1 plays a protective role in preclinical models of vascular injury [37,38] and exerts anti-inflammatory effects [20].

Our study shows that HF is associated with reduced ANGPT1 plasma concentrations, increased ANGPT2 levels, and an increased ANGPT2/ANGPT1 ratio as compared with healthy controls. Of importance, different alterations of ANGPT1 and ANGPT2 expression have been detected in patients with IHF and NIHF. For instance, plasma levels of ANGPT1 are significantly decreased only in NIHF, but not in IHF patients compared to controls. Contrarywise, circulating levels of ANGPT2 are increased in NIHF, but not in IHF patients compared to healthy donors. Moreover, the ANGPT2/ANGPT1 ratio, an index of vascular permeability [63], was exclusively increased only in NIHF patients.

We did not find a correlation between plasma concentrations of ANGPT1 or ANGPT2 and BNP in either IHF or NIHF patients. In contrast, a recent study reported a significant correlation between serum concentrations of ANGPT2 and NT-proBNP in more than 200 patients that had undergone diagnostic cardiac catheterization [73]. Several explanations can justify these apparently different results. The latter study included patients with (54%) or without coronary artery disease, as well as with comorbidities (e.g., diabetes, hypertension) that may have influenced the results. In our study, the population of IHF and NIHF participants was selectively included, and patients with comorbidities were not selected. Although the two examined cohorts were rather small, the patients examined in our study were very homogeneous for the principal clinical and demographic features.

Our results may have clinical implications for patients suffering from HF. First, if confirmed in larger cohorts, the evaluation of plasma concentrations of ANGPT1, ANGPT2, and their ratio may be useful in the identification of different pathophysiological patterns underlying ischemic and non-ischemic HF. Second, the unique role of the ANGPTs/Tie2 signaling pathway in vascular stability suggests that it could serve as a target for therapeutic intervention in diseases whose pathophysiology comprises the alteration of vascular integrity [27], such as HF. Recently, it has been demonstrated that ANGPT2 inhibition, through an anti-ANGPT2 blocking antibody, substantially alleviated autoimmune inflammation [70]. Importantly, specific ANGPT2 deletion or the use of an anti-ANGPT2 antibody markedly reduced cardiac hypoxia, proinflammatory macrophage polarization, adverse vascular remodeling, and the consequent progression of HF after myocardial infarction in mice [15]. The results of the latter study contribute to elucidating the roles of ANGPT2 in the pathogenesis of post-ischemic cardiovascular remodeling. Finally, these fascinating experimental results designate ANGPT2 as a promising therapeutic target to prevent/ameliorate HF.

VEGF-A is a powerful permeability factor [42] and a potent proangiogenic and proinflammatory mediator [41,74]. Although several clinical studies have found elevated circulating levels of VEGF-A in myocardial infarction [28,47,48,49,50], the role of this mediator in HF still remains poorly elucidated. Our results show that, differently from acute vascular injuries, plasma levels of VEGF-A are not altered in the overall HF population or in either IHF or NIHF patients. Thus, our results suggest that this mediator could play different roles in an acute vs. chronic setting of myocardial ischemia.

VEGF-C and VEGF-D are major lymphangiogenic factors produced by human macrophages [52,75] and cardiac mast cells [43]. In a mouse model of HF, VEGF-C and VEGF-D were upregulated in the early stages of disease, with levels returning afterwards to baseline [76]. Levels of VEGF-C have been reported as elevated in patients with ischemic or non-ischemic cardiomyopathy [77]. An increased level of VEGF-D was found in an animal model of ischemic cardiomyopathy [78] as well as in human atherosclerotic lesions [79]. Recent evidence indicates that lymphangiogenesis [66] and VEGF-C improve cardiac functions after experimental myocardial infarction [80]. Our results indicate that the plasma concentrations of VEGF-C were similar in patients with HF (IHF and NIHF) and controls. Interestingly, the circulating concentrations of VEGF-D were increased in HF patients compared to controls, but significant differences were exclusively detected in IHF patients. The differential alterations of VEGF-C and VEGF-D in these patients is intriguing but not surprising. In fact, recent evidence demonstrates that VEGF-C and VEGF-D can differently modulate the immune system [45]. The possible role of VEGF-D in HF patients deserves further investigations.

PLA_2_ activity was found increased in plasma from both groups of HF patients compared to healthy controls. PLA_2_ modulates endothelial cell migration and vascular permeability in vitro and in humans [53,55,56,57,81]. Previous studies have demonstrated that circulating levels of sPLA_2_ predict coronary events in patients with coronary artery disease [58], as well as in apparently healthy men and women [59]. Moreover, serum sPLA_2_ levels also predicts readmission for HF after myocardial infarction [60]. More recently, elevated circulatory levels of sPLA_2_ were associated with risk of early atherosclerosis [62]. Our study is, to our knowledge, the first to demonstrate that high plasma concentrations of PLA_2_ activity can be found in HF patients, both with an ischemic and non-ischemic etiology.

Along with the epidemiologic transition of global population, the pathophysiology of HF has changed over time. According to the Framingham Heart Study, hypertension represented the most frequently associated condition in HF patients, irrespective of LVEF [82]. It is widely recognized that coronary heart disease represents the predominant cause of HFrEF [83]. Coronary stenosis-dependent cardiomyocyte hypoxia, through inadequate oxygen supply to metabolic needs and leading to ventricular dysfunction, may be the result of both acute and chronic cardiac ischemia. Indeed, in acute coronary syndromes, a sudden drop in myocardial perfusion rapidly determines cardiomyocyte injury. In the setting of chronic ischemia there is often an imbalance between coronary blood flow and augmented demand due to progressive atherosclerosis, especially under stress. This leads over time to hibernation, stunning, and secondary myocardial remodeling, resulting in reduced cardiac output [84].

NIHF pathophysiology is more heterogeneous due to several etiologic factors that are sometimes concurrent. The most relevant causes of NIHF are represented by primary valvular diseases, arterial hypertension, microbial cardiomyopathy, DM, toxic agents (drugs or alcohol), and genetic cardiomyopathies. Once all the listed factors have been excluded, idiopathic HF is the resulting diagnosis [85]. In NIHF there is a primary injury in the cardiomyocyte structure and function that manifests in cell apoptosis and a consequent substitution with fribotic tissue, without alteration in coronary flow.

However, independently from ischemic or non-ischemic etiology, all patients suffering from systolic HF present reduction of LVEF, maladaptive LV remodeling, and similar clinical presentations including dyspnea and hydro-saline retention. Our results identify an HF-dependent impact on the expression levels of several vascular permeability and inflammatory mediators, with different patterns in the clinical setting of NIHF and IHF that potentially reflect the above-mentioned pathophysiological differences.

Several immune cells produce sPLA_2_ [86,87,88], ANGPTs [15,17,18,24,70,89], VEGF-A [43,52,74,90], and VEGF-C/VEGF-D [52,74,91]. In this study, we did not address the issue of the contribution of different cells to the increased plasma levels of these powerful inflammatory mediators observed in patients with IHF or NIHF. ANGPT2 appears to be a potential therapeutic option in experimental heart failure [15]. Future studies with the aim of identifying the cellular sources of these powerful mediators could lead to the identification of novel and selective therapeutic targets in IHF and NIHF patients.

The limited number of subjects enrolled represented the main limitation of the present investigation. However, it is important to point out that in order to identify specific differences between NIHF and IHF the study protocol included stringent exclusion criteria to reduce potential interference with the inflammatory and angiogenic patterns explored in the study. Indeed, very common comorbidities such as COPD, DM, immune disorders, malignancies, and severe obesity were excluded from the study. As a consequence, the patients examined were very homogeneous, but rather small. The results of this preliminary study will have to be extended in a future multicenter trial examining larger cohorts of IHF and NIHF patients.

## 5. Conclusions

In the present study we demonstrated that the ANGPT system is selectively modulated in NIHF patients, with an increased ANGPT2/ANGPT1 ratio compared to IHF and controls, whereas VEGF-D was exclusively augmented in IHF patients. In contrast, sPLA2 activity was increased in both IHF and NIHF patients compared to healthy controls. To the best of our knowledge this represents the first evidence reporting that several regulators of vascular permeability and inflammation is specifically altered in patients with IHF and NIHF, paving the way for the identification of new molecular mechanisms underlying HF pathophysiology and novel therapeutic targets.

## Figures and Tables

**Table 1:** Demographic and clinical characteristics of patients with ischemic heart failure (IHF) or non-ischemic heart failure (NIHF) and healthy controls.

Characteristics	Healthy Controls (*N* = 42)	IHF (*N* = 19)	NIHF (*N* = 25)
Age-median years (range)	75.5 (46–98)	77 (54–87)	65 (45–87)
Gender male-no. (%)	16 (38.1)	12 (63.1)	16 (64)
BMI (kg/m^2^)	25.2 ± 4.1	25.4 ± 3.0	25.5 ± 4.2
Caucasian (%)	100	100	100
BNP (pg/mL)	50.6 ± 32.0	1025.8 ± 733.3 *	968.6 ± 802.2 *
Leukocytes (×10^3^/mm^3^)	7.2 ± 2.5	8.6 ± 4.1	7.9 ± 3.0
GFR (mL/min)	71.2 ± 23.3	48.5 ± 24.3	69.6 ± 32.4
LVEF (%)	61.6 ± 5.8	34.3 ± 6.9 *	34.6 ± 7.4 *

Data are expressed as the mean ± standard deviation of the mean (BMI, BNP, Leukocytes, GFR, LVEF) or median value (Age). IHF: ischemic heart failure; NIHF: non-ischemic heart failure; BNP: B-type natriuretic peptide; GFR: glomerular filtration rate (assessed through CKD-EPI equation); LVEF: left ventricular ejection fraction. * *p* < 0.01 when compared to healthy controls analyzed by one-way ANOVA and Bonferroni’s multiple comparison test.

## Abbreviations

ACE	angiotensin-converting enzyme
ANGPT	angiopoietin
ARB	angiotensin receptor blocker
BMI	body mass index
BNP	B-type natriuretic peptide
COPD	chronic obstructive pulmonary disease
DM	diabetes mellitus
DOPC	dioleoylphosphatidylcholine
DOPG	dioleoyphosphatidylglycerol
EF	ejection fraction
GFR	glomerular filtration rate
HF	heart failure
IHF	ischemic heart failure
LVEF	left ventricular ejection fraction
NIHF	non-ischemic heart failure
PLA_2_	phospholipase A_2_
VEGF	vascular endothelial growth factor
VEGFR	vascular endothelial growth factor receptor
